# Correction: Co-infection and ICU-acquired infection in COVID-19 ICU patients: a secondary analysis of the UNITE-COVID data set

**DOI:** 10.1186/s13054-022-04124-8

**Published:** 2022-08-17

**Authors:** Andrew Conway Morris, Katharina Kohler, Thomas De Corte, Ari Ercole, Harm-Jan De Grooth, Paul W. G. Elbers, Pedro Povoa, Rui Morais, Despoina Koulenti, Sameer Jog, Nathan Nielsen, Alasdair Jubb, Maurizio Cecconi, Jan De Waele, for the ESICM UNITE COVID investigators, Marco Bezzi, Alicia Gira, Philipp Eller, Tarikul Hamid, Injamam Ull Haque, Wim De Buyser, Antonella Cudia, Daniel De Backer, Pierre Foulon, Vincent Collin, Jan De Waele, Jolien Van Hecke, Elisabeth De Waele, Claire Van Malderen, Jean-Baptiste Mesland, Michael Piagnerelli, Lionel Haentjens, Nicolas De Schryver, Jan Van Leemput, Philippe Vanhove, Pierre Bulpa, Viktoria Ilieva, David Katz, Anna Geagea, Alexandra Binnie, Fernando Tirapegui, Gustavo Lago, Jerónimo Graf, Rodrigo Perez-Araos, Patricio Vargas, Felipe Martinez, Eduardo Labarca, Daniel Molano Franco, Daniela Parra-Tanoux, Luis Felipe Reyes, David Yepes, Filip Periš, Sanda Stojanović Stipić, Cynthia Vanessa Campozano Burgos, Paulo Roberto Navas Boada, Jose Luis Barberan Brun, Juan Pablo Paredes Ballesteros, Ahmed Hammouda, Omar Elmandouh, Ahmed Azzam, Aliae Mohamed Hussein, Islam Galal, Ahmed K. Awad, Mohammed A. Azab, Maged Abdalla, Hebatallah Assal, Mostafa Alfishawy, Sherief Ghozy, Samar Tharwat, Abdullah Eldaly, Veronika Reinhard, Anne Chrisment, Chrystelle Poyat, Julio Badie, Fernando Berdaguer Ferrari, Björn Weiss, Karl Friedrich Kuhn, Julius J. Grunow, Marco Lorenz, Stefan Schaller, Peter Spieth, Marc Bota, Falk Fichtner, Kristina Fuest, Tobias Lahmer, Johannes Herrmann, Patrick Meybohm, Nikolaos Markou, Georgia Vasileiadou, Evangelia Chrysanthopoulou, Panagiotis Papamichalis, Ioanna Soultati, Sameer Jog, Kushal Kalvit, Sheila Nainan Myatra, Ivan Krupa, Aisa Tharwat, Alistair Nichol, Aine McCarthy, Ata Mahmoodpoor, Tommaso Tonetti, Paolo Isoni, Savino Spadaro, Carlo Alberto Volta, Lucia Mirabella, Alberto Noto, Gaetano Florio, Amedeo Guzzardella, Chiara Paleari, Federica Baccanelli, Marzia Savi, Massimo Antonelli, Barbara Vaccarini, Giorgia Montrucchio, Gabriele Sales, Katia Donadello, Leonardo Gottin, Enrico Polati, Silvia De Rosa, Demet Sulemanji, Abdurraouf Abusalama, Muhammed Elhadi, Montelongo Felipe De Jesus, Daniel Rodriguez Gonzalez, Nancy Canedo, Alejandro Esquivel Chavez, Tarek Dendane, Bart Grady, Ben de Jong, Eveline van der Heiden, Patrick Thoral, Bas van den Bogaard, Peter E. Spronk, Sefanja Achterberg, Melanie Groeneveld, Ralph K. L. So, Calvin de Wijs, Harm Scholten, Albertus Beishuizen, Alexander D. Cornet, Auke C. Reidinga, Hetty Kranen, Roos Mensink, Sylvia den Boer, Marcel de Groot, Oliver Beck, Carina Bethlehem, Bas van Bussel, Tim Frenzel, Celestine de Jong, Rob Wilting, Jozef Kesecioglu, Jannet Mehagnoul-Schipper, Datonye Alasia, Ashok Kumar, Ahad Qayyum, Muhammad Rana, Mustafa Abu Jayyab, Rosario Quispe Sierra, Aaron Mark Hernandez, Lúcia Taborda, Tiago Ramires, Catarina Silva, Carolina Roriz, Pedro Póvoa, Patricia Patricio, Maria Lurdes Santos, Vasco Costa, Pedro Cunha, Celina Gonçalves, Sandra Nunes, João Camões, Diana Adrião, Ana Oliveira, Ali Omrani, Muna Al Maslamani, Abdurrahmaan Suei elbuzidi, Bara Mahmoud Al qudah, Abdel Rauof Akkari, Mohamed Alkhatteb, Anas Baiou, Ahmed Husain, Mohamed Alwraidat, Ibrahim Abdulsalam Saif, Dana Bakdach, Amna Ahmed, Mohamed Aleef, Awadh Bintaher, Cristina Petrisor, Evgeniy Popov, Ksenia Popova, Mariia Dementienko, Boris Teplykh, Alexey Pyregov, Liubov Davydova, Belskii Vladislav, Elena Neporada, Ivan Zverev, Svetlana Meshchaninova, Dmitry Sokolov, Elena Gavrilova, Irena Shlyk, Igor Poliakov, Mapинa Bлacoвa, Ohoud Aljuhani, Amina Alkhalaf, Felwa Bin Humaid, Yaseen Arabi, Ahmed Kuhail, Omar Elrabi, Madihah Alghnam, Amit Kansal, Vui Kian Ho, Jensen Ng, Raquel Rodrígez García, Xiana Taboada Fraga, Mª del Pilar García-Bonillo, Antonio Padilla-Serrano, Marta Martin Cuadrado, Carlos Ferrando, Ignacio Catalan-Monzon, Laura Galarza, Fernando Frutos-Vivar, Jorge Jimenez, Carmen Rodríguez-Solis, Enric Franquesa-Gonzalez, Guillermo Pérez Acosta, Luciano Santana Cabrera, Juan Pablo Aviles Parra, Francisco Muñoyerro Gonzalez, Maria del Carmen Lorente Conesa, Ignacio Yago Martinez Varela, Orville Victoriano Baez Pravia, Maria Cruz Martin Delgado, Carlos Munoz de Cabo, Ana-Maria Ioan, Cesar Perez-Calvo, Arnoldo Santos, Ane Abad-Motos, Javier Ripolles-Melchor, Belén Civantos Martin, Santiago Yus Teruel, Juan Higuera Lucas, Aaron Blandino Ortiz, Raúl de Pablo Sánchez, Jesús Emilio Barrueco-Francioni, Lorena Forcelledo Espina, José M. Bonell-Goytisolo, Iñigo Salaverria, Antonia Socias Mir, Emilio Rodriguez-Ruiz, Virginia Hidalgo Valverde, Patricia Jimeno Cubero, Francisca Arbol Linde, Nieves Cruza Leganes, Juan Maria Romeu, Pablo Concha, José Angel Berezo-Garcia, Virginia Fraile, Cristina Cuenca-Rubio, David Perez-Torres, Ainhoa Serrano, Clara Martínez Valero, Andrea Ortiz Suner, Leire Larrañaga, Noemi Legaristi, Gerardo Ferrigno, Safa Khlafalla, Rosita Bihariesingh-Sanchit, Frank Zoerner, Jonathan Grip, Kristina Kilsand, Jonas Österlind, Magnus von Seth, Johan Berkius, Samuele Ceruti, Andrea Glotta, Seval Izdes, Işıl Özkoçak Turan, Ahmet Cosar, Burcin Halacli, Necla Dereli, Mehmet Yilmaz, Türkay Akbas, Gülseren Elay, Selin Eyüpoğlu, Yelíz Bílír, Kemal Tolga Saraçoğlu, Ebru Kaya, Ayca Sultan Sahin, Pervin Korkmaz Ekren, Tuğçe Mengi, Kezban Ozmen Suner, Yakup Tomak, Ahmet Eroglu, Asad Alsabbah, Katie Hanlon, Kevin Gervin, Sean McMahon, Samantha Hagan, Caroline V. Higenbottam, Randeep Mullhi, Lottie Poulton, Tomasz Torlinski, Allen Gareth, Nick Truman, Gopal Vijayakumar, Chris Hall, Alasdair Jubb, Lenka Cagova, Nicola Jones, Sam Graham, Nicole Robin, Amanda Cowton, Adrian Donnelly, Natalia Singatullina, Melanie Kent, Carole Boulanger, Zoë Campbell, Elizabeth Potter, Natalie Duric, Tamas Szakmany, Orinta Kviatkovske, Nandor Marczin, Caroline Ellis, Rajnish Saha, Chunda Sri-Chandana, John Allan, Lana Mumelj, Harish Venkatesh, Vera Nina Gotz, Anthony Cochrane, Nuttha Lumlertgul, Barbara Ficial, Susan Jain, Giulia Beatrice Crapelli, Aikaterini Vlachou, David Golden, Sweyn Garrioch, Jeremy Henning, Gupta Loveleena, Miriam Davey, Lina Grauslyte, Erika Salciute-Simene, Martin Cook, Danny Barling, Phil Broadhurst, Sarah Purvis, Spivey Michael, Benjamin Shuker, Irina Grecu, Daniel Harding, Natalia Singatullina, James T. Dean, Nathan D. Nielsen, Sama Al-Bayati, Mohammed Al-Sadawi, Mariane Charron, Peter Stubenrauch, Jairo Santanilla, Catherine Wentowski, Dorothea Rosenberger, Polikseni Eksarko, Randeep Jawa

**Affiliations:** 1 Division of Anaesthesia, Department of Medicine, Level 4 Addenbrooke’s Hospital, University of Cambridge, Hills Road, Cambridge, UK; 2 Division of Immunology, Department of Pathology, University of Cambridge, Cambridge, UK; 3 JVF Intensive Care Unit, Addenbrooke’s Hospital, Cambridge, UK; 4 Department of Intensive Care Medicine, Ghent University Hospital, Ghent, Belgium; 5 Dept of Internal Medicine and Pediatrics, Faculty of Medicine and Health Sciences, Ghent University, Ghent, Belgium; 6 Neurocritical Care Unit, Addenbrooke’s Hospital, Cambridge, UK; 7 Department of Intensive Care, Amsterdam UMC Location Vrije Universiteit Amsterdam, Amsterdam, The Netherlands; 8 Laboratory for Critical Care Computational Intelligence, Amsterdam Medical Data Science, Amsterdam UMC, Vrije Universiteit, Amsterdam, The Netherlands; 9 Nova Medical School, New University, Lisbon, Portugal; 10 Center for Clinical Epidemiology and Research Unit of Clinical Epidemiology, OUH Odense University Hospital, Odense, Denmark; 11 Polyvalent Intensive Care Unit, Hospital de São Francisco Xavier, CHLO, Lisbon, Portugal; 12 2Nd Critical Care Department, Attikon University Hospital, University of Athens, Athens, Greece; 13 UQ Centre for Clinical Research (UQCCR), Faculty of Medicine, The University of Queensland, Brisbane, Australia; 14 Deenanath Mangeshkar Hospital and Research Center, Pune, India; 15 Divisions of Pulmonary, Critical Care and Sleep Medicine and Transfusion Medicine, University of New Mexico School of Medicine, Albuquerque, NM USA; 16 Department of Anaesthesia, Humanitas University, Milan, Italy

## Correction to: Critical Care (2022) 26:236 10.1186/s13054-022-04108-8

Following publication of the original article [1], an error was identified in the article title: COVID‑19 was incorrectly captured as COIVD‑19. The article title has been updated above and in the original article.
